# A Difficult-to-Diagnose Collision Tumor in a Patient With Gastric-Type Duodenal Cancer and Gastric Cancer: A Report of a Rare Case

**DOI:** 10.7759/cureus.92278

**Published:** 2025-09-14

**Authors:** Takahiro Haruna, Ryuji Ohashi, Junji Ueda, Daigo Yoshimori, Yoshinobu Shioda, Hiroshi Yoshida

**Affiliations:** 1 Department of Gastroenterological Surgery, Nippon Medical School, Tokyo, JPN; 2 Department of Integrated Diagnostic Pathology, Nippon Medical School, Tokyo, JPN

**Keywords:** collision tumor, gastric cancer, gastric-type duodenal cancer, lymph node dissection, lymph node metastasis

## Abstract

Collision tumors, characterized by the coexistence of two distinct malignancies within the same area, are extremely rare. Herein, we report a collision tumor that was difficult to diagnose preoperatively in a patient with gastric cancer and gastric-type duodenal cancer. An 82-year-old woman with a history of melena and anemia was diagnosed with progressive gastric cancer of the gastric antrum at the lesser curvature. Part of the tumor infiltrated the pyloric ring but was not detected in the duodenal bulb within the observable range. Enhanced computed tomography revealed wall thickening from the gastric antrum up to the pyloric ring, with swelling of the lesser curvature and a subpyloric lymph node. The patient underwent a distal gastrectomy and D2 lymph node dissection for gastric cancer. Pathological findings showed gastric cancer (papillary adenocarcinoma and moderately differentiated tubular adenocarcinoma) positive for mucin 5AC (MUC5AC), and duodenal cancer (solid-type poorly differentiated adenocarcinoma > non-solid-type poorly differentiated adenocarcinoma) positive for MUC5AC and mucin 6 (MUC6), with both tumors meeting at the pyloric ring. Lymph node metastasis from gastric and duodenal cancer was also detected. No previous reports of collision tumors involving both gastric-type duodenal cancer and gastric cancer exist. In patients with gastric cancer with duodenal invasion, the possibility of duodenal cancer complications and the extent of lymph node dissection should be considered.

## Introduction

A collision tumor is defined as the coexistence of two adjacent but histologically distinct tumors with no histologic admixture [1]. These morphologically separated tumors are sharply demarcated from each other at the same site. Such a tumor is distinct from neoplasms that demonstrate heterologous or mixed elements. It is extremely rare and usually found randomly during pathological evaluation of surgically resected specimens [1-3], and more frequently encountered in the cranium, lung, gastroesophageal junction, liver, rectum, bladder, and uterus [4].

Duodenal cancer is essentially considered a cancer arising in the non-ampullary parts of the duodenum. Non-ampullary duodenal cancer (NADC) is rare, representing less than 0.5% of all gastrointestinal malignancies and approximately 45% of small bowel adenocarcinomas. There are no established treatment guidelines, and surgical resection is known to be the only curative treatment. Lymph node dissection is important in terms of accurate staging and local disease control, and contributes to improved prognosis [5-7]. In this report, we describe a case of collision tumor caused by NADC and advanced gastric cancer, which has not been previously reported.

## Case presentation

**Figure 1:**
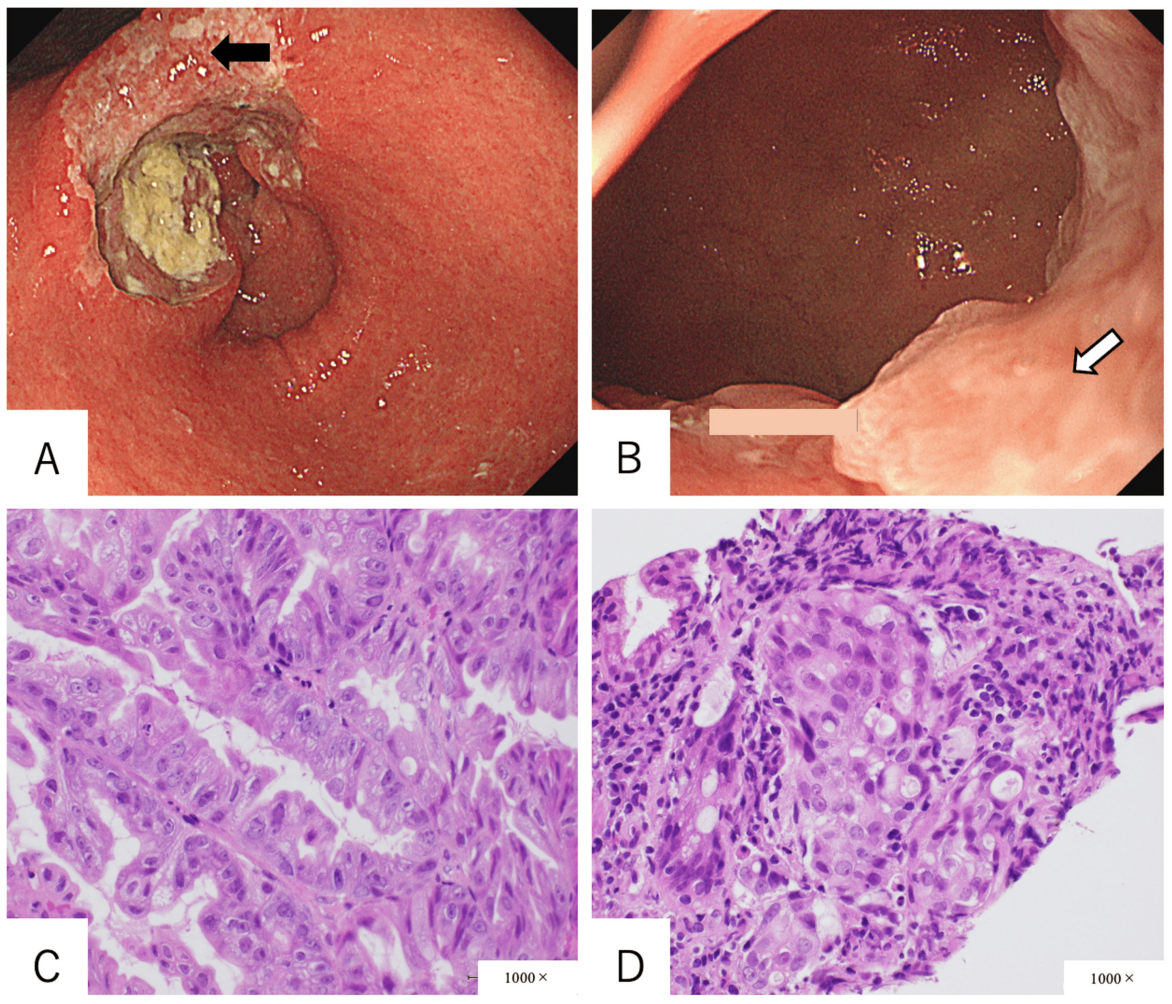
(A) Endoscopic finding demonstrating a gastric type 3 tumor centered on the lesser curvature of the antrum. (B) Endoscopic finding demonstrating gastric tumor invasion to the pyloric ring. (C) Histopathological finding on the proximal side of the tumor demonstrating poorly differentiated adenocarcinoma (black arrow in Figure 1A). (D) Histopathological finding demonstrating moderately differentiated adenocarcinoma above the pyloric ring (white arrow in Figure 1B).

An 82-year-old woman with a history of hypertension, hyperlipidemia, and *Helicobacter pylori* eradication was referred to our hospital because of lightheadedness and anemia. She was afebrile, with a heart rate of 70 beats per minute, blood pressure of 118/83 mmHg, and oxygen saturation of 100% on room air. Laboratory investigations revealed a hemoglobin level of 6.6 g/dL. She underwent an emergency upper gastrointestinal endoscopy, which revealed a large type 3 tumor centered on the lesser curvature of the gastric antrum. The tumor had invaded the gastric angle on the proximal side and up to the pyloric ring on the antral side (Figures 1A, 1B). No obvious invasion into the duodenal bulb was detected on endoscopy. Biopsy revealed a poorly differentiated adenocarcinoma on the proximal side and moderately differentiated tubular adenocarcinoma above the pyloric ring (Figures 1C, 1D).

**Figure 2:**
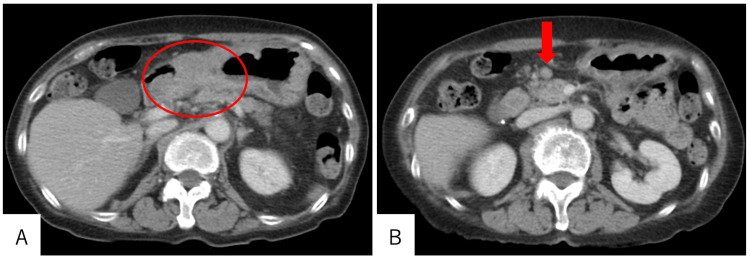
(A-B) Enhanced computed tomography finding demonstrating wall thickening of the gastric antrum (red circle) and significant lymph node enlargement near the pyloric ring (red arrow).

Enhanced computed tomography revealed wall thickening of the gastric antrum and enlarged lymph nodes near the pyloric ring (Figure 2). Distant metastasis was not observed. These findings confirmed the diagnosis of advanced gastric cancer (cT3N1M0, stage Ⅲ) with duodenal invasion. The patients underwent an open distal gastrectomy, D2 lymph node dissection, and Roux-en-Y reconstruction. The operative time was 233 minutes, and the intraoperative blood loss was 48 mL. No postoperative complications were observed, and the patient was discharged on postoperative day eight.

**Figure 3:**
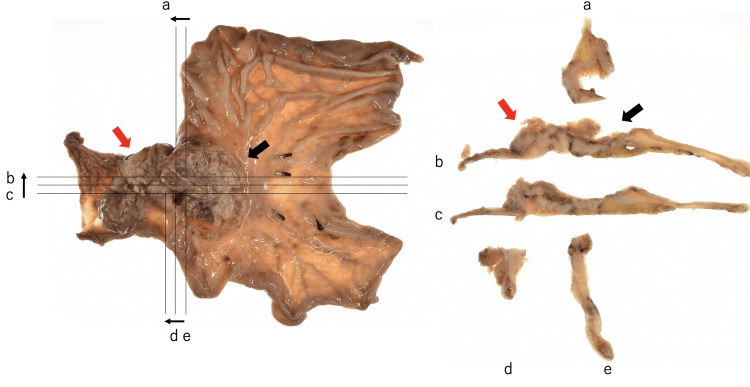
Macroscopic finding demonstrating a type 3 tumor occupying the gastric antecubital area (black arrow) and a type 1 tumor localizing from the pyloric ring to the duodenal bulb (red arrow). The right side of the image is the proximal side, and the left side is the distal side. The letters of the alphabet apply to each of the cut-out sections and their cross-sections.

Macroscopic examination revealed a type 3 tumor occupying the gastric antecubital area and a type 1 tumor localized from the pyloric ring to the duodenal bulb. These tumors converged at the pyloric ring, a feature that differed from the preoperative endoscopic findings (Figure 3).

**Figure 4:**
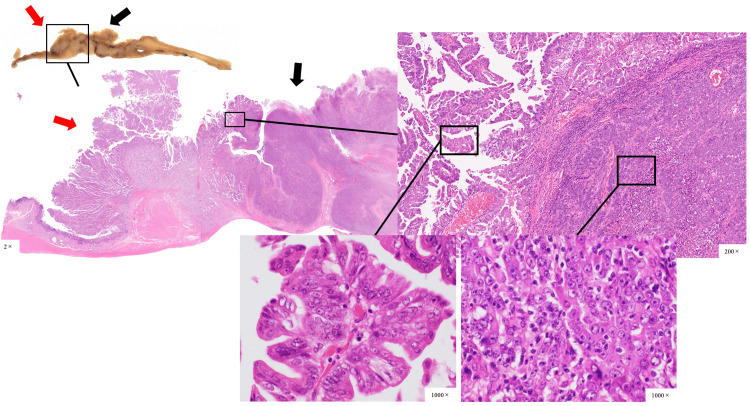
Histopathological finding of hematoxylin and eosin stain demonstrating type 3 tumor (black arrow) was composed mainly of poorly differentiated adenocarcinomas, and type 1 tumor (red arrow) was composed mainly of moderately differentiated adenocarcinomas, and the two tumors were clearly demarcated.

Histopathological findings showed that the type 3 tumor was mainly composed of poorly differentiated adenocarcinomas, while the type 1 tumor was mainly composed of moderately differentiated adenocarcinomas, and the two tumors were clearly demarcated (Figure 4).

**Figure 5:**
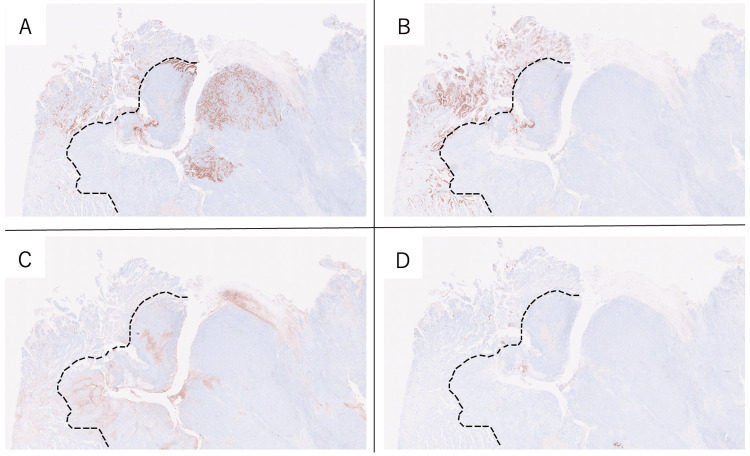
(A-D) The right side of the image is the proximal side of gastric cancer, and the left side is the distal side of duodenal cancer. (A) Immunostaining histopathological findings demonstrating MUC5AC staining in the gastric cancer and duodenal cancer, (B) MUC6 staining only in the duodenal cancer, and (C) no CD10 or (D) MUC2 staining in these cancers.

The type 1 tumor was diagnosed as duodenal cancer because they were mainly located in the duodenum in the pyloric ring and Brunner's gland. Therefore, this case was diagnosed as a collision tumor between gastric cancer (L, Gre, type 3, 50 mm × 50 mm, solid type poorly differentiated adenocarcinoma > non-solid type poorly differentiated adenocarcinoma, pT4 (SE), INFb, Ly1b, V0, pPM0 (53mm), pDM0 (25mm), pN2 (3/32)) and duodenal cancer (D (bulb), type 1, 31 mm × 52 mm, papillary adenocarcinoma, moderately differentiated tubular adenocarcinoma > mucinous adenocarcinoma, pT3 (SS), INFb, Ly0, V0, pPM0 (53 mm), pDM0 (25 mm), pN2 (3/32)). Among the subpyloric lymph node metastases, one lymph node metastasis was of gastric origin and the other of duodenal origin. Immunostaining histopathological examinations showed mucin 5AC (MUC5AC) positivity in both gastric and duodenal cancers, mucin 6 (MUC6) positivity in only duodenal cancer, and CD10 and MUC2 negative staining for both cancers (Figure 5). Therefore, the duodenal cancer was classified as a gastric type.

## Discussion

NADC can be classified into two main categories according to their phenotype: intestinal and gastric type [8]. Intestinal-type NADC are found more frequently on the distal side of Vater’s papilla, while gastric-type NADC are found frequently on the proximal side of Vater’s papilla, with characteristic background changes, such as foveolar hyperplasia and heterotopic fundic glands [8,9]. Although intestinal-type NADC are more frequently observed than gastric-type NADC, the ratio of adenocarcinomas to intestinal-type NADC is lower than that to gastric-type NADC. Therefore, most gastric-type cases involve advanced cancer and lymph node metastasis and have a poor prognosis [8-10]. Our case showed MUC5AC and MUC6 staining in the duodenal cancer. MUC5AC stains foveolar epithelial cells in normal gastric mucosa, and MUC6 stains glandular mucosa cells in normal gastric mucosa and Brunner’s glands. Therefore, it is possible that the duodenal cancer might be a pyloric gland-type gastric cancer originating from the heterotopic fundic gland, unlike cancers of chronic inflammatory origin.

Surgical resection of the primary tumor is the only curative treatment option for NADC because there is no standard chemotherapy regimen for treating NADCs [11]. In particular, pancreaticoduodenectomy with regional lymphadenectomy should be performed as an oncologically adequate option in patients with advanced NADC, regardless of the tumor location [12]. Moreover, adequate regional lymphadenectomy for advanced tumors located in the duodenal first portion was recommended in the Union for International Cancer Control (UICC) tumor, node, and metastasis (TNM) classification [13]. The frequency of metastasis in suprapyloric (No. 5), infrapyloric (No. 6), common hepatic artery (No. 8), hepatoduodenal (No. 12), posterior pancreaticoduodenal (No. 13), superior mesenteric artery (No. 14), and anterior pancreaticoduodenal (No. 17) lymph nodes has been reported as very high, with a frequency ranging from 9.1% to 50.0% [7,12-15]. The five-year survival rate in duodenal cancers with lymph node metastases is 19.2%-51.7% [12]. Our patient had advanced duodenal cancer with lymph node metastasis (No. 6). Preoperative diagnosis was very difficult because the duodenal carcinoma was predominantly located near the pyloric ring and overlapped with the gastric carcinoma invasion. However, if an accurate preoperative diagnosis of duodenal cancer was possible, the appropriate oncological procedure in this case might have been pancreaticoduodenectomy with a complicated resection of gastric cancer. In such cases, regional lymph node dissection for gastric cancer would also have been required, which would have been a highly invasive procedure [16]. For an elderly patient, the balance between radicality involving overall prognosis and invasiveness must be considered. Thus, when gastric cancer with suspected pyloric invasion is suspected, multiple biopsies should be obtained separately from both the gastric side and the duodenal side of the pyloric ring. Identifying different histological types could raise suspicion for a collision tumor with duodenal cancer and alter the surgical planning.

Collision tumors associated with NADC are extremely rare. Only four case reports of collision tumors have been published so far: duodenal neuroendocrine cancer (NEC) and gastric adenocarcinoma (GAC); duodenal carcinoid and pancreatic cancer; duodenal adenocarcinoma (DAC) and duodenal NEC; and duodenal neuroendocrine tumor and GAC (Table 1) [1,17-19]. However, to the best of our knowledge, no cases of DAC and GAC have been reported. Although the DAC and duodenal NEC case was found in the third portion of the duodenum, other cases were found from the gastric antrum to the first portion of the duodenum. None of the collision tumors could be diagnosed preoperatively, as in our case, and the pathological findings were used to diagnose the collision tumors. Two pancreaticoduodenectomies, a distal gastrectomy, and an endoscopic submucosal dissection were performed. In all cases, the appropriate surgical procedure was selected for the level of tumor progression, and the prognosis was good.

**Table 1:** Clinical features of five collision tumors. NEC: neuroendocrine cancer; GAC: gastric adenocarcinoma; DG: distal gastrectomy; PD: pancreaticoduodenectomy; DAC: duodenal adenocarcinoma; NET: neuroendocrine tumor; ESD: endoscopic submucosal dissection.

Author	Age/sex	Clinical feature	Location	Histopathological diagnosis	Treatment
Fukui et al. (2001) [17]	63/M	Nausea	Gastric antrum and duodenum bulb	Duodenal NEC, GAC	DG
Ferrando Marco et al. (2007) [18]	64/M	Epigastric pain	Duodenum and head of the pancreas	Duodenal carcinoid, pancreatic adenocarcinoma	PD
Peng et al. (2012) [1]	52/M	Weight loss, postprandial vomiting	Third portion of the duodenum	DAC, duodenal NEC	PD
Kaneko et al. (2016) [19]	78/F	Nausea, right hypochondrial pain	Gastric antrum and duodenum bulb	Duodenal NET, GAC	ESD
Our case (2023)	82/F	Lightheadedness	Gastric antrum and duodenum bulb	DAC, GAC	DG

## Conclusions

To the best of our knowledge, this is the first report of a collision tumor involving gastric-type DAC and GAC. In this report, the occurrence of gastric-type DAC and the treatment of gastric-type DAC when it can be diagnosed preoperatively are discussed. Accurate preoperative diagnosis for DAC is important because effective chemotherapy has not been established. As previously reported, the diagnosis of collision tumors appears to be difficult. In patients with gastric cancer with duodenal invasion, the possibility of duodenal cancer complications and the extent of lymph node dissection should be considered.
